# Liver Diseases: From Bench to Bedside

**DOI:** 10.3390/ijms25105454

**Published:** 2024-05-17

**Authors:** Tatsuo Kanda, Reina Sasaki-Tanaka, Shuji Terai

**Affiliations:** 1 Division of Gastroenterology and Hepatology, Uonuma Institute of Community Medicine, Niigata University Medical and Dental Hospital, Minamiuonuma 949-7302, Japan; 2 Division of Gastroenterology and Hepatology, Graduate School of Medical and Dental Sciences, Niigata University, Niigata 951-8520, Japan; reina_sasaki_0925@yahoo.co.jp (R.S.-T.); terais@med.niigata-u.ac.jp (S.T.)

The human genome encodes at least 500 protein kinases, and among them, there are at least 90 tyrosine kinases [1]. Of the 90 human tyrosine kinases, 58 and 32 are receptor-type and non-receptor-type families, respectively [2]. Receptor-type family includes anaplastic lymphoma kinase (ALK), formyl peptide receptor 2 (FPR2/ALX), discoidin domain receptor tyrosine kinase 1 (DDR1/DDR), epidermal growth factor receptor (EGFR), erythropoietin-producing hepatocellular carcinoma kinase receptor (EPH), fibroblast growth factor receptor (FGFR), insulin receptor (INSR), MET proto-oncogene, receptor tyrosine kinase (MET), muscle-associated receptor tyrosine kinase (MUSK), platelet-derived growth factor receptor (PDGFR), protein tyrosine kinase 7 (PTK7), RET proto-oncogene (RET), receptor tyrosine kinase-like orphan receptor (ROR), ROS proto-oncogene 1, receptor tyrosine kinase (ROS), receptor-like tyrosine kinase (RYK), vascular endothelial growth factor receptor (VEGFR/FLT1), and apoptosis associated tyrosine kinase (AATYK). The non-receptor-type family at least includes ABL proto-oncogene 1, non-receptor tyrosine kinase (ABL), tyrosine kinase non-receptor 2 (TNK2/ACK), C-terminal Src kinase (CSK), protein tyrosine kinase 2 (PTK2/FAK), FES proto-oncogene, tyrosine kinase (FES), fyn-related Src family tyrosine kinase (FRK), Janus kinase (JAK), SRC proto-oncogene, non-receptor tyrosine kinase (SRC/c-Src), tec protein tyrosine kinase (TEC), and spleen associated tyrosine kinase (SYK) [2]. Certain molecules and their signaling pathways are targets of systemic therapies using tyrosine kinase inhibitors (TKIs) for cancers, including hepatocellular carcinoma [3,4]. During the treatment of TKIs, attention should be paid to the adverse events, such as hypertension, hypothyroidism, skin reactions, proteinuria, etc. [4].

Drug repurposing is increasingly becoming an attractive modality because it avoids risky compounds without the higher overall development costs and longer development of the drugs [5]. Drug repurposing of preexisting drugs for the treatment of hepatitis A virus (HAV) infection has been reported [6]. In this Special Issue, Sasaki-Tanaka et al. [7] examined the in vitro anti-HAV activity of 1134 US Food and Drug Administration (FDA)-approved drugs and finally found that masitinib, one of the TKIs, suppresses HAV replication, although further studies in vivo will be needed for clinical use.

Masitinib has a good safety profile at 7.5 mg/kg daily. Masitinib can increase overall survival and slow Amyotrophic Lateral Sclerosis (ALS) Functional Rating Scale-Revised (ALSFRS-R) deterioration among patients with ALS, which is a neurodegenerative disease with high mortality and morbidity rates affecting both upper and lower motor neurons [8]. Masitinib has also been used in the treatment of various cancers or systemic mastocytosis because it is a novel TKI for numerous targets, including c-Kit (CD117), PDGFR, and FGFR [9,10,11].

Although TKIs are generally used in the treatment of malignant diseases, there are several reports that TKIs could inhibit hepatitis A virus (HAV), hepatitis B virus (HBV), hepatitis C virus (HCV), and hepatitis E virus (HEV) replication in vivo and/or in vitro (Table 1) [12,13,14,15,16,17,18,19,20].

The JAK inhibitors SD-1029, AG490, and AZD1480, which reduce La protein expression, could inhibit HAV internal ribosomal entry-site-mediated translation and HAV replication [12]. The 5 nM SB202190 also inhibited HAV genotype IIIA HA 11-1299 replication in human hepatoma cell lines [13].

Of interest, geldanamycin or herbimycin A could block HBV replication [14]. Sorafenib, one of the TKIs used for advanced hepatocellular carcinoma, inhibits HBV and HCV replication [15,16,17,18]. Schlevogt et al. [19] observed the occurrence of chronic HEV infection after treatment with the Bruton’s tyrosine kinase inhibitor ibrutinib in vivo. Thus, some TKIs showed efficacy for the inhibition of hepatitis viral replication and cellular cytotoxicity to some extent (Table 1).

Although contrary opinions exist [20,21,22,23], careful attention should be paid to the reactivation and chronicity of hepatitis virus infection during the treatment of TKIs. Some TKIs should be useful for the control of hepatitis viral replication. TKIs also play a role in non-malignant diseases [24].

Imamura et al. [25] reported that the Src/c-Abl signaling pathway may be a potentially useful target for developing new drugs to treat ALS. They developed a phenotypic screen to repurpose existing drugs using ALS motor neuron survival, using motor neurons that were generated from induced pluripotent stem cells (iPSCs) derived from an ALS patient with a mutation on superoxide dismutase 1 (SOD1). They found that bosutinib, which is one of the tyrosine kinase inhibitors to treat chronic myeloid leukemia (CML) [26], modestly extended the survival of a mouse model of ALS with an SOD1 mutation. A Phase I dose escalation study of bosutinib was performed for ALS patients [27]. In this way, drug repurposing of TKIs to treat other diseases than malignant diseases is promising.

Diagnosis and treatment for liver diseases, including extrahepatic manifestations, have been progressing recently [28,29]. Although nucleos(t)ide analogues and direct-acting antivirals are currently available and effective for HBV and HCV-related liver diseases, more effective and more convenient drugs may be needed for hepatitis virus infection. Drug repurposing of TKIs to treat hepatitis virus infection seems like one of the most hopeful and promising methods.

## Figures and Tables

**Table 1:** Various tyrosine kinase inhibitors (TKIs) could inhibit the replication of hepatitis viruses.

Hepatitis Viruses	TKIs	Authors (Years) [References]
HAV	SD-1029, AG490, and AZD1480	Kanda et al. (2015) [12]
HAV	SB202190	Kanda et al. (2021) [13]
HAV	Masitinib	Sasaki-Tanaka et al. (2023) [7]
HBV	Geldanamycin and herbimycin A	Bouchard et al. (2003) [14]
HBV	Sorafenib	Wang et al. (2021) [15]
HCV	Sorafenib	Bürckstümmer et al. (2006) [16] Himmelsbach et al. (2009) [17] Descamps et al. (2015) [18]
HEV	Ibrutinib	Schlevogt et al. (2019) [19]

HAV, hepatitis A virus; HBV, hepatitis B virus; HCV, hepatitis C virus; and HEV, hepatitis E virus.
